# Seroprevalence of SARS-CoV-2 in Croatian solid-organ transplant recipients

**DOI:** 10.11613/BM.2021.030901

**Published:** 2021-10-15

**Authors:** Anna Mrzljak, Željka Jureković, Jadranka Pavičić-Šarić, Vladimir Stevanović, Irena Tabain, Željka Hruškar, Danko Mikulić, Ljubo Barbić, Tatjana Vilibić-Čavlek

**Affiliations:** 1 Department of Gastroenterology and Hepatology, University Hospital Center Zagreb, Zagreb, Croatia; 2 School of Medicine, University of Zagreb, Zagreb, Croatia; 3 Transplant Centre, Merkur University Hospital, Zagreb, Croatia; 4 Department of Microbiology and Infectious Diseases with Clinic, Faculty of Veterinary Medicine, University of Zagreb, Zagreb, Croatia; 5 Department of Virology, Croatian Institute of Public Health, Zagreb, Croatia

**Keywords:** COVID-19, seroprevalence, solid-organ transplant recipients, SARS-CoV-2 antibodies

## Abstract

**Introduction:**

The data on the coronavirus disease (COVID-19) in solid-organ transplant recipients (SOTRs) in Croatia is unknown. The aim of this study was to analyze the seroprevalence of severe acute respiratory syndrome coronavirus 2 (SARS-CoV-2) in Croatian SOTRs.

**Materials and methods:**

From 7 September to 27 November 2020 (beginning of the second COVID-19 pandemic wave), a cross-sectional screening for COVID-19 was performed in the adult outpatient liver (LTRs; N = 280) and kidney transplant recipients (KTRs; N = 232). Serum samples were initially tested for SARS-CoV-2 IgG antibodies using a commercial enzyme-linked immunosorbent assay (ELISA; Vircell Microbiologists, Granada, Spain). All positive samples were confirmed using a virus neutralization test (VNT). Data on risk exposure and COVID-19 related symptoms were collected using a questionnaire.

**Results:**

The transplanted cohort’s seroprevalence detected by ELISA and VNT was 20.1% and 3.1%, respectively. Neutralizing (NT) antibodies developed in 15.6% of anti-SARS-CoV-2 ELISA IgG positive SOTRs. The difference in seropositivity rates between LTRs and KTRs was not statistically significant (ELISA 21.1% *vs*. 19.0%, P = 0.554; VNT 3.6% *vs*. 2.6%, P = 0.082). Overall VNT positivity rates were higher in patients who reported participation in large community events (5.9% *vs*. 1.0%; P = 0.027) as well as in patients who reported COVID-19 related symptoms in the past six months. In addition, symptomatic VNT positive patients showed significantly higher (P = 0.031) NT antibody titers (median 128, interquartile range (IQR) = 32-128) compared to asymptomatic patients (median 16, IQR = 16-48).

**Conclusions:**

This study showed that 15.6% of anti-SARS-CoV-2 ELISA positive Croatian SOTRs developed NT antibodies indicating protective immunity. Further studies are needed to determine the dynamic of NT antibodies and COVID-19 immunity duration in immunocompromised populations such as LTRs and KTRs.

## Introduction

Patients after solid-organ transplantation have been severely affected by the coronavirus disease 2019 (COVID-19) pandemic. Disruption of medical services, higher risk of infection, and its consequences are major reasons for the concern in this immunocompromised group of patients. Immunosuppression modulates the immune system, affecting humoral immune responses against various pathogens, including viruses. The serologic immunoglobulin G (IgG) response against severe acute respiratory syndrome coronavirus 2 (SARS-CoV-2) has been an intense investigation area; however, the immune response to SARS-CoV-2 after transplantation remains unknown and directs further research. The screening with SARS-CoV-2 IgG enzyme-linked immunosorbent assay (ELISA) followed by virus neutralization test (VNT) confirmation is a reliable approach for seroepidemiological studies, which is crucial to assess infection attack rates in the population and herd immunity (*1*). Currently, there is a lack of seroprevalence data for specific patient populations, such as transplanted patients. In Croatia, the first case of COVID-19 has been reported on 25 February 2020, affecting more than 334,229 people and resulting in 7130 deaths by 2 May 2021. During the first (February-May 2020) and second (August 2020-February 2021) pandemic waves, cases have been recorded in all Croatian counties. However, there are few published studies on the prevalence of SARS-CoV-2 in selected population groups and the COVID-19 seroprevalence in the solid-organ transplant recipients (SOTRs) is unknown. In this study, we analyzed the seroprevalence of SARS-CoV-2 at the beginning of the second pandemic wave in liver (LTRs) and kidney transplant recipients (KTRs) in Croatia.

## Materials and methods

### Subjects

We performed a cross-sectional screening for COVID-19 in 512 adult outpatient SOTRs in a single transplant center in Croatia: LTRs (N = 280) and KTRs (N = 232) from 7 September to 27 November 2020, at the beginning of the second wave COVID-19 epidemic curve in Croatia. All transplanted patients who had an appointment in the transplant outpatient clinic in this period and signed the informed consent were included in the study. All patients were asymptomatic at the time of sampling. The study was approved by the Hospital Ethics Committee (reference: 03/1-7732/9).

### Methods

Each patient was submitted to a COVID-19 questionnaire regarding the risk exposure (attendance to large meetings such as weddings/funerals/concerts, traveling abroad or receiving blood products) and COVID-19 related symptoms (fever, cough, ageusia, anosmia, headache, and myalgia) since the beginning of 2020, as well as previous SARS-CoV-2 reverse transcription polymerase chain reaction (RT-PCR) testing. The questionnaire response rate was 82%. All participants were enrolled in the study, regardless of the response to questionnaire. Blood samples were collected by venipuncture in serum blood tubes (without anticoagulants) in a non-fasting state at any time of day. After centrifugation (3000 rpm for 10 min), serum samples were stored at - 20 °C until testing.

All samples were initially screened for the presence of SARS-CoV-2 IgG antibodies. A commercial enzyme-linked immunosorbent assay (ELISA; COVID-19 ELISA IgG, Vircell Microbiologists, Granada, Spain) was used to detect SARS-CoV-2 IgG antibodies in human serum samples. The assay is based on recombinant spike glycoprotein (S) and nucleocapsid protein (N) antigens of SARS-CoV-2. The results were calculated and expressed as antibody index [AI = (sample optical density (OD)/cut off serum mean OD)] x 10 and interpreted as follows: < 4 negative, 4-6 borderline, > 6 positive. Positive, negative, and cut-off controls have been run with each test run. Serologic tests were run on automated ELISA analyzer (VirClia, Vircell Microbiologists, Granada, Spain). Initial ELISA validation was performed using serum samples from patients with RT-PCR confirmed COVID-19 (N = 15) collected 4-34 days after disease onset and asymptomatic persons (N = 15) with negative RT-PCR and negative VNT (*2*).

All ELISA reactive samples were confirmed using a VNT. The SARS-CoV-2 isolated in Vero E6 cells from the nasopharyngeal swab of a COVID-19 patient was used for the VNT. The maximum cytopathic effect was visible on the 4th day, and the virus replication was confirmed by RT-PCR. Virus titer (TCID_50_) was calculated using the Reed and Muench formula (*3*). Heat-inactivated serum samples (56 °C/30 min) were tested in duplicate in 96-well plates. Two-fold serum dilutions starting from 1:2 were prepared and mixed with the equal-volume (25 μL) suspension containing median tissue culture infectious dose (100 TCID_50_) of the virus. After 1 h of incubation (37 °C) in a CO_2_ incubator, a 50 μL suspension of Vero E6 cells (final concentration: 2 x10^5^ cells/mL) was added to each well and incubated for 4 days. To ensure optimal testing results, virus antigen used in each run was back titrated and positive sample with known titer as well as negative control sample were included in each plate. The antibody titer was defined as the highest serum dilution’s reciprocal value that showed 100% neutralization in at least half of the infected wells. Neutralizing (NT) antibody titers ≥ 8 were considered positive (*4*).

### Statistical analysis

Statistical analysis was performed using SPSS Version 17.0.1. (SPSS Inc, Chicago, IL, USA). Differences between the groups were tested using χ^2^ or Fisher’s exact test (categorical variables) and Mann-Whitney U test (ordinal or numerical variables).The level of statistical significance was set at P < 0.05.

## Results

The investigated cohort included 232 KTRs (median 53 years, 22-80; male 60.8%) and 280 LTRs (median 63 years, 22-83, male 72.5%). Out of 512 transplanted patients, 103 or 20.1% (95%CI = 16.4-26.3) were anti-SARS-CoV-2 IgG positive using ELISA (AI > 6). The difference in IgG seroprevalence between LTRs and KTRs was not statistically significant (N = 59 or 21.1%; 95%CI = 16.4-26.3 *vs*. N = 44 or 19.0%; 95%CI = 14.1-24.6, P = 0.554). Out of 103 seropositive participants, only 20.4% reported previous nasopharyngeal (NP) swab RT-PCR testing, with 6/21 confirmed as SARS-CoV-2 positives. One anti-SARS-CoV-2 IgG negative patient reported a previous positive RT-PCR test.

The overall prevalence of NT antibodies (titer ≥ 8) was 3.1% (95%CI = 1.8-5.0), with no statistically significant difference (P = 0.536) between KTRs (2.6%; 95%CI = 0.9-5.5) and LTRs (3.6%; 95%CI = 1.7-6.5). The prevalence of NT antibodies in ELISA positive patients was 16/103 (15.5%, 95%CI = 9.1-24.0).

Seroprevalence rates according to risk factors and clinical symptoms in KTRs and LTRs who responded to a questionnaire (N = 420) are presented in Tables 1 and 2. There were no differences in the overall VNT positivity rate in SOTRs regarding received blood products (3.0%; 95%CI = 0.1-12.0 *vs*. 1.6%; 95%CI = 0.6-3.4, P = 0.553) and travelling habits (5.3%; 95%CI = 0.6-17.8 *vs.* 1.1%; 95%CI = 0.3-2.7, P = 0.085). However, a significant difference was found regarding attendance to large group meetings (5.9%; 95%CI = 0.7-19.7 *vs.* 1.0%; 95%CI = 0.3-2.6, P = 0.027).

**Table 1:** COVID-19 seroprevalence in kidney transplant recipients according to potential risk factors and clinical symptoms (N = 207)

		**N (%) tested**	**ELISA IgG positive**			**VNT positive**		
			**N (%)**	**95%CI**	**P**	**N (%)**	**95%CI**	**P**
**Risk factors**								
Blood transfusion								
Yes		11 (5.3)	1 (9.1)	0.2-41.3	0.593	0 (0)	0-28.5*	1.000
No		196 (94.7)	10 (5.6)	2.8-9.8		6 (3.1)	1.1-6.5	
Participation in large community events								
Yes		16 (7.7)	4 (25.0)	7.2-52.4	0.463	2 (12.5)	1.5-38.3	0.082
No		191 (92.3)	31 (16.2)	11.3-22.2		4 (2.1)	0.6-5.3	
Travelling abroad								
Yes		28 (13.5)	5 (17.8)	6.1-36.9	0.853	2 (7.1)	0.8-23.5	0.200
No		179 (86.5)	29 (16.2)	11.1-22.4		4 (2.2)	0.6-5.6	
**Clinical symptoms**								
Fever								
Yes		30 (14.5)	9 (30.8)	14.7-49.4	0.095	5 (16.6)	5.6-34.7	< 0.001
No		177 (85.5)	26 (14.7)	9.8-20.8		1 (0.6)	0-3.1	
Cough								
Yes		21 (10.2)	6 (28.6)	11.3-52.2	0.1224	5 (23.8)	8.2-47.1	< 0.001
No		186 (89.8)	29 (15.6)	10.7-21.6		1 (0.5)	0-2.9	
Breathing difficulties								
Yes		16 (7.7)	3 (18.7)	4.0-45.6	0.864	1 (6.3)	0.2-30.2	0.396
No		191 (92.3)	32 (16.7)	11.7-22.8		5 (2.6)	0.8-6.0	
Anosmia								
Yes		5 (2.4)	4 (80.0)	28.3-99.5	0.009	4 (80.0)	28.4-99.5	< 0.001
No		202 (97.6)	31 (15.3)	10.6-21.1		2 (1.0)	0.1-3.5	
Ageusia								
Yes		4 (1.9)	3 (75.0)	19.4 - 99.3	0.030	3 (75.0)	19.4-99.3	< 0.001
No		203 (98.1)	32 (15.8)	11.0 - 21.5		3 (1.5)	0.3-4.3	
Headache								
Yes		25 (12.1)	5 (20.0)	6.8-40.7	0.714	1 (4.0)	0.1-20.3	0.546
No		182 (87.9)	30 (16.5)	11.4-22.7		5 (2.7)	0.9-6.3	
Muscle aches								
Yes		15 (7.2)	4 (26.6)	11.2-22.1	0.395	2 (13.3)	1.6-40.5	0.074
No		192 (92.8)	31 (16.1)	7.8-55.1		4 (2.1)	0.5-5.3	
*One-sided 97.5% confidence interval. ELISA - enzyme-linked immunosorbent assay. IgG - immunoglobulin G. VNT - virus neutralization test. P < 0.05 was considered statistically significant.								

**Table 2:** COVID-19 seroprevalence in liver transplant recipients according to potential risk factors and clinical symptoms (N = 213)

	**N (%)** **tested**	**ELISA IgG positive**			**VNT positive**		
		**N (%)**	**95%CI**	**P**	**N (%)**	**95%CI**	**P**
**Risk factors**							
Blood transfusion							
Yes	33 (15.5)	6 (18.2)	7.0-35.5	0.889	1 (3.0)	0.1-15.8	0.496
No	180 (84.5)	35 (19.4)	13.9-26.0		3 (1.1)	0.2-3.1	
Participation in large community events							
Yes	18 (8.5)	4 (22.2)	0.6-47.6	0.785	1 (5.6)	0.1-27.3	0.242
No	195 (91.5)	37 (19.0)	13.7-25.2		2 (1.0)	0.1-3.7	
Travelling abroad							
Yes	10 (4.7)	4 (40.0)	12.2-73.8	0.194	2 (20.0)	2.5-55.6	0.015
No	203 (95.3)	37 (18.2)	13.2-24.2		2 (1.0)	0.1-3.5	
**Clinical symptoms**							
Fever							
Yes	34 (15.9)	11 (32.3)	17.4-50.5	0.095	2 (5.9)	0.7-19.7	0.129
No	179 (84.1)	30 (16.7)	11.6-23.1		2 (1.1)	0.1-4.0	
Cough							
Yes	19 (8.9)	2 (10.5)	1.3-33.1	0.390	2 (10.5)	1.3-33.1	0.047
No	194 (91.1)	39 (20.1)	14.7-26.4		2 (1.0)	0.1-3.7	
Breathing difficulties							
Yes	13 (6.1)	1 (7.7)	0.2-36.0	0.347	3 (23.1)	0.5-53.8	0.001
No	200 (93.9)	40 (0.2)	14.7-20.2		1 (0.5)	0-2.7	
Anosmia							
Yes	2 (0.9)	0 (0)	0-8.4*	0.534	0 (0)	0 - 84.1*	1.000
No	211 (99.1)	41 (19.4)	14.3-25.4		4 (1.9)	0.5 - 4.8	
Ageusia							
Yes	6 (2.8)	1(16.6)	0.4-64.1	0.892	0 (0)	0-45.9*	1.000
No	207 (97.2)	40 (19.3)	14.2-25.4		4 (1.9)	0.5-4.8	
Headache							
Yes	29 (13.6)	2 (6.9)	0.8-22.8	0.118	0 (0)	0-11.9*	1.000
No	184 (86.4)	39 (21.2)	15.6-27.9		4 (2.2)	0.6-5.5	
Muscle aches							
Yes	25 (11.7)	4 (16.0)	4.5-36.1	0.708	1 (4.0)	0.1-20.3	0.402
No	188 (88.3)	37 (19.7)	14.3-26.2		3 (1.6)	0.3-4.6	
*One-sided 97.5% confidence interval. ELISA - enzyme-linked immunosorbent assay. IgG - immunoglobulin G. VNT - virus neutralization test. P < 0.05 was considered statistically significant.							

Anti-SARS-CoV-2 IgG antibodies were found in both asymptomatic patients (N = 301) and patients who reported COVID-19 related symptoms in the past six months (N = 119). In the KTR group, significantly higher VNT positivity rates were documented in patients who reported fever, cough, anosmia and ageusia compared to patients who reported no symptoms (Table 1). In a LTR group, NT antibodies were more frequently detected in patients who reported cough and breathing difficulties (Table 2).

**Figure 1:**
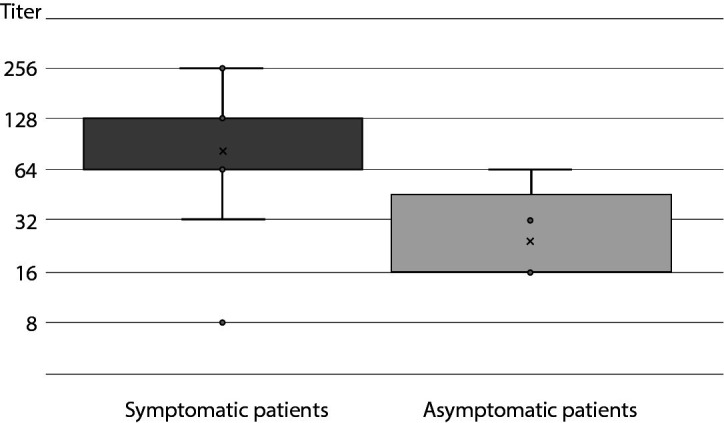
Anti-SARS-CoV-2 neutralizing antibody titers in symptomatic (N = 11, dark) and asymptomatic (N = 5, light) transplanted patients (P = 0.031).

Symptomatic patients had significantly higher (P = 0.031) NT antibody titers (median 128, IQR = 32-128) compared to asymptomatic patients (median 16, IQR = 16-48) (Figure 1).

## Discussion

This is the first study to demonstrate the anti-SARS-CoV-2 seroprevalence in the Croatian LTRs and KTRs. The study was conducted at the beginning of the second COVID-19 pandemic wave (September-November 2020), demonstrating the transplanted cohort’s seroprevalence of 20.1% using ELISA with 15.6% of anti-SARS-CoV-2 IgG positive SOTR who developed NT antibodies. The seroprevalence in this population group was found to be significantly higher compared to some other populations. At the end of the first wave, the SARS-CoV-2 seroprevalence in Croatia was very low. Using immunochromatography assay, IgG antibodies were detected in only 1.27% of industry workers Split-Dalmatia and Šibenik-Knin County (*5*). Besides, 2.7% of healthcare workers tested seropositive using ELISA, while NT antibodies were found in only 1.5% of the tested (*4*). Additionally, 2.2% of the general population were shown to be ELISA positive after the first wave, with low titers of NT antibodies detected in only 0.2% of individuals (*6*). A more recent study conducted among children from Children’s hospital Zagreb showed that the prevalence of anti-SARS-CoV-2 antibodies differed significantly between the first wave (2.9%) and the second wave (8.4%) of the COVID-19 pandemic (*7*).

The presentation of SOTRs with COVID-19 appears similar to the general population, and the majority develop symptoms such as fever, cough, or diarrhea; however, some patients may remain asymptomatic (*8*, *9*). As in our cohort, anti-SARS-CoV-2 IgG was found in both symptomatic and asymptomatic SOTRs, with significant difference in the VNT positivity among patients who reported COVID-19 related symptoms in the past six months (KTRs - fever, cough, anosmia and ageusia; LTRs - cough and breathing difficulties) compared to the asymptomatic patients.

In the context of immunocompromised populations, such as SOTRs, an open question remains regarding the robust antibody response after the SARS-CoV-2 infection. In June 2020, a UK study demonstrated an anti-SARS-CoV-2 IgG prevalence of 10.4% in a large cohort of KTRs. Interestingly, 33% of patients with prior COVID-19 infection were IgG seronegative at a median time of 36 days post-diagnosis (*10*). Prendecki *et al.* tested historical pre- and post-COVID-19 samples of 38 KTR, showing that all pre-COVID-19 samples were negative while 68.4%, 92.1%, and 81.6% of post-COVID-19 samples tested antibody positive using three different serological assays. Notably, 7.9% of patients did not have detectable antibodies on any assay, representing a seroconversion failure (*10*).

An American study found that KTRs are capable of developing an immune response to SARS-CoV-2 at least early on, similar to those observed in the general population (*11*). A recent study showed that 51% of adult SOTRs had positive anti-nucleocapsid antibodies at least 7 days after the diagnosis of SARS-CoV-2 infection and that KTRs were less likely to test positive compared to other SOTRs. Transplant-related variables (time after the transplant and immunosuppression) were important predictors of antibody response to SARS-CoV-2 infection (*12*).

Patients with more severe disease tend towards higher antibody levels (*9*). Similar to the results of other studies, our study showed that symptomatic patients developed higher NT antibody titers compared to asymptomatic ones. Compared to a previous Croatian study in the general population, the prevalence of NT antibodies was slightly higher in the SOTRs (3.1% *vs.* 2.2%) (*6*). However, it is important to note that NT antibodies were detected in 15.6% ELISA positive SOTRs compared to 8.3% of the general population. The study in the Croatian general population was conducted after the first wave, while SOTRs were tested at the beginning of the second wave when the number of COVID-19 cases increased sharply which could explained at least in part the observed difference in VNT positivity rates.

It was previously shown that most of the asymptomatic and mildly ill patients did not produce significant levels of IgM antibodies, indicating that the IgM diagnosis is not sensitive and efficient. In contrast, similar IgG responses were detected in all patients (*13*). Therefore, IgM antibodies were not tested in this study and should be regarded as one of the limitations of the study.

In addition, the study’s cross-sectional design precludes us from drawing conclusions regarding the duration of anti-SARS-CoV-2 IgG in SOTRs or distinguishing between impaired antibody production or a rapid decline, as we lack the data regarding the level of the immunosuppression and serial time point measurements with longer follow-up.

In immunocompromised patients such as SOTRs, it is necessary to understand the immune response to SARS-CoV-2 and identify factors associated with inadequate antibody response among those who failed to seroconvert. Therefore, further studies are needed to assess the dynamic of NT antibodies and COVID-19 immunity duration in immunocompromised populations such as LTRs and KTRs.
